# Direct oral anticoagulants vs. low-molecular-weight heparin for pulmonary embolism in patients with glioblastoma

**DOI:** 10.1007/s10143-021-01539-9

**Published:** 2021-04-26

**Authors:** Daniel Dubinski, Sae-Yeon Won, Martin Voss, Fee Keil, Wolfgang Miesbach, Bedjan Behmanesh, Max Dosch, Peter Baumgarten, Joshua D. Bernstock, Volker Seifert, Thomas M. Freiman, Florian Gessler

**Affiliations:** 1 Department of Neurosurgery, University Hospital, Goethe University, Schleusenweg 2-16, 60598 Frankfurt, Germany; 2 Dr. Senckenberg Institute of Neurooncology, Goethe University Hospital, Frankfurt, Germany; 3 Institute of Neuroradiology, University Hospital, Goethe University, Frankfurt, Germany; 4 Department of Hemostaseology and Transfusion Medicine, University Hospital, DRK-Blutspendedienst Baden-Württemberg-Hessen gGmbH, Frankfurt, Germany; 5 Department of Neurosurgery, Birgham and Women’s, Harvard Medical School, Boston, MA USA

**Keywords:** Pulmonary embolism, Direct oral anticoagulation, Low-molecular-weight heparin, Therapeutic anticoagulation, Glioblastoma survival

## Abstract

Glioblastoma (GBM) is a cancer type with high thrombogenic potential and GBM patients are therefore at a particularly high risk for thrombotic events. To date, only limited data on anticoagulation management after pulmonary embolism (PE) in GBM is available and the sporadic use of DOACs remains off-label. A retrospective cohort analysis of patients with GBM and postoperative, thoracic CT scan confirmed PE was performed. Clinical course, follow-up at 6 and 12 months and the overall survival (OS) were evaluated using medical charts and neuroradiological data. Out of 584 GBM patients, 8% suffered from postoperative PE. Out of these, 30% received direct oral anticoagulants (DOACs) and 70% low-molecular-weight heparin (LMWH) for therapeutic anticoagulation. There was no significant difference in major intracranial hemorrhage (ICH), re-thrombosis, or re-embolism between the two cohorts. Although statistically non-significant, a tendency to reduced mRS at 6 and 12 months was observed in the LMWH cohort. Furthermore, patients receiving DOACs had a statistical benefit in OS. In our analysis, DOACs showed a satisfactory safety profile in terms of major ICH, re-thrombosis, and re-embolism compared to LMWH in GBM patients with postoperative PE. Prospective, randomized trials are urgent to evaluate DOACs for therapeutic anticoagulation in GBM patients with PE.

## Introduction

Venous thromboembolism (VTE) is a feared and well-described complication in the perioperative setting of cancer patients in general and is aggravated by several factors such as venous stasis, hemiparesis, damage to the endothelia, and release of tissue factor (TF) in GBM patients in specific [5]. If manifest, VTE patients require immediate intensive care treatment with pharmacological and/or mechanical intervention. The clinical presentation can vary from asymptomatically wo unspecific symptoms (dyspnea, chest pain, anxiety) to cardiac arrest due to acute heart failure [13]. For GBM patients, the cumulative probability of VTE ranges between 20 and 30% per year of survival and is the highest during the postoperative setting but remains higher than other malignancies throughout the course of the disease [3, 8, 16]. The key measurements of PE prevention comprise mechanical and pharmacological thromboprophylaxis [15]. In systemic cancer patients, LMWH is nowadays preferred over vitamin K antagonists (VKA) [4, 10]. Furthermore, in systemic cancer, DOACs (factor Xa and thrombin inhibitors) have been found non-inferior to LMWH with respect to recurrent VTE, but the rate of major bleeding was higher with DOACs than with LMWH [10]. However, DOACs are in wide use for patients requiring therapeutic anticoagulation, mainly due to their ease of use and the lack of monitoring requirements. However, no prospective randomized trial is available for GBM patients with the necessity to therapeutic anticoagulation. Therefore, no preferred anticoagulant-regiment is established to date and the initiation of DOACs for PE after GBM remains explicit off-label [6, 11]. We conducted this analysis with a particular focus on the clinical course and outcome of GBM patients with PE and different anticoagulants.

## Methods

### Patients and data collection

For this retrospective analysis, an ethical approval was obtained from the ethics committee of the University Hospital Frankfurt, Germany (Identification number: 20–683). As a non-interventional single-center study, no patient consent was necessary. All patients who underwent craniotomy for tumor resection and had the radiologically confirmed diagnosis of PE from 2010 to 2019 were added to the institutional SPSS-database (version 20, SPSS, IBM Inc., Armonk, NY). Treatment decisions, including determination for surgery, were rendered by the local interdisciplinary tumor board. Patient follow-up was achieved in the outpatient neurosurgical department. Patients modified Rankin Scale (mRS) was ascertained by an attending neurosurgeon. Included data on patient characteristics and clinical course were evaluated through the chart record. Exclusion criteria were the presence of pre-existing hematological disorders (factor V deficiency, hemophilia, thrombocythemia, von-Willebrand syndrome etc.), the lack of cranial MRI, thoracic CT scan, or incomplete follow-up chart.

### Postoperative thrombosis prophylaxis

After craniotomy and tumor resection, patients were transmitted to the institution’s neurosurgical intensive care unit (NICU). Prophylactic anticoagulation was initiated 10 h postoperatively with 1 × 20 mg Clexane® and increased up to 1 × 40 mg Clexane® from the first postoperative day onwards. Before transferring to a regular ward, patients were mobilized with the assistance of a physiotherapist or a NICU-nurse where possible. Furthermore, all patients were urged to wear thrombosis stockings.

### PE diagnostics and management

Indication for thoracic CT scan was the acute onset of one, or the combination of the following symptoms: collapse upon mobilization, shock, hypotonia, tachycardia, dyspnea, chest pain, or dip in oxygen saturation [1]. Thoracic CT scans were performed in the department of neuroradiology at a multidetector Philips CT Scanner by an attending neuroradiologist. Ultravist® 300 was administered intravenously (80 ml/kg, 4.0 ml/s) and imaging started after the contrasting of the pulmonary artery. Upon radiological confirmation of PE, anticoagulation was initiated with weight adapted Clexane® and peripheral blood samples analyzing anti-X-a serum levels obtained until values in the range of 0.5–1 IU/ml were reached. After PE onset, screening for DVT was performed by one experienced specialist in angiology via Doppler ultrasound of the lower extremities. Further change of therapeutic anticoagulation regime was realized by experienced specialists at the department of hemostaseology at the authors’ institution around day 14 after craniotomy. As standardized treatment protocols are currently lacking, the choice of the anticoagulant for each patient was at the doctor’s discretion.

### Patients follow-up

The postoperative period was defined as the time from operation to discharge from neurosurgery. After discharge, patient’s follow-up was carried out in the department of neurooncology every 3 months until the transfer to palliative care or hospice. The response assessment in neurooncology (RANO) criteria was conducted by an attending neuroradiologist. In detail, cranial MRI including standard sequences (T1-weighted (w), T2-w, T2*-w, FLAIR, diffusion-weighted imaging (DWI), T1-w with contrast agent) and a T1-w sequence with contrast agent was performed. Major intracranial hemorrhage (ICH) was defined as any hemorrhage that was ≥ 10 ml in volume, required surgical intervention, or was associated with clinical symptoms, such as nausea and vomiting, or focal neurologic deficit [12]. Anticoagulation was continued for 6 months after PE. If clinical suspicion of re-thrombosis was present, Doppler ultrasound of the lower extremities were performed by one experienced specialist in angiology.

### Statistics

Data analysis was performed with IBM SPSS Statistics Version 23.0 (SPSS Inc., IBM Corp., Armonk, NY, USA). For patients and tumor characteristics, descriptive statistics were used. Fisher’s exact test was used for the comparison of categorical variables between the cohorts. For continuous parameters, the Wilcoxon-Mann–Whitney test was used. To assess the impact of the variables, odds ratio (OR) with 95% confidence intervals (CI) were calculated. Results with *p* ≤ 0.05 were considered statistically relevant. To estimate the survival rates, the Kaplan–Meier analysis was used. The differences between curves were assessed using the log-rank test. Overall survival (OS) was defined as the time of first presentation to death.

## Results

**Fig. 1:**
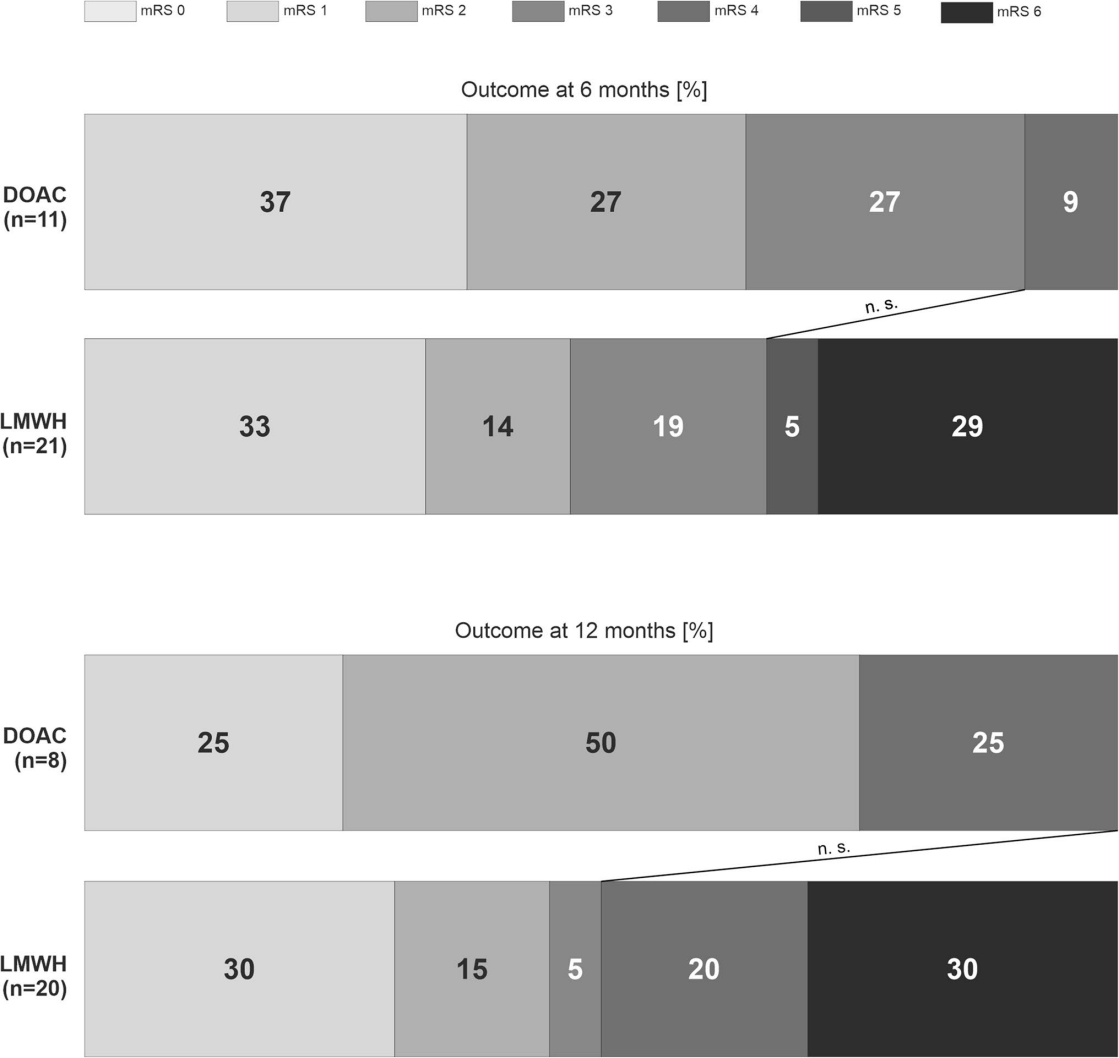
Functional outcome via modified Rankin Scale in patients with pulmonary embolism and glioblastoma according to their anticoagulation regime. Abbreviations: mRS, modified Rankin Scale; DOAC, direct oral anticoagulants; LMWH, low-molecular-weight heparin

**Fig. 2:**
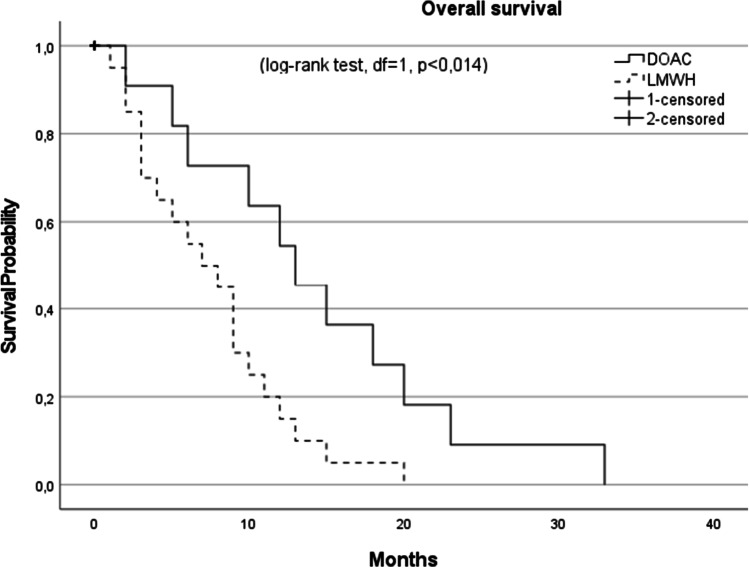
Kaplan–Meier plot after pulmonary embolism diagnosis. The median survival for patients receiving DOACs (solid line) was 15 months compared with 9 months for those receiving LMWH (dashed line). Abbreviations: DOAC, direct oral anticoagulants; LMWH, low-molecular-weight heparin

In total, 46 patients with PE after GBM resection were included. Fourteen patients received a DOAC, whereas 32 received LMWH. The distributions of DOACs were as follows: rivaroxaban (Xarelto®), *n* = 6 and Edoxaban (Lixiana®), *n* = 8. Both cohorts were well matched for age and sex (0 = 0.49 and 0.42 respectively) (Table 1). Although statistically non-significant, the LMWH cohort had a higher incidence of coronary heart disease, tobacco abuse, hypertension, hypercholesterinemia, and diabetes mellitus (*p* = 0.15, 0.22, 1, 1, and 0.22 respectively). The medication at admission between both cohorts was statistically non-significant; however, the LMWH cohort had a higher amount of platelet aggregation inhibitors (*p* = 0.17) (Table 1). Neither did the time from craniotomy to PE nor to discharge differs between the cohorts. The accomplishment of gross total resection did not differ between the cohorts (*p* = 0.10). Neither did the admission nor discharge status displayed in KPS and mRS differs between the cohorts (*p* = 1 and *p* = 0.49, respectively). The clinical course was similar in both groups with one resuscitation in each group. No histopathological difference was observed in terms of MGMT promotor methylation and IDH-1 mutation status (*p* = 0.20 and *p* = 0.49). Postoperative re-bleeding and the need for urgent re-operation were present in one patient of the DOAC cohort and two patients in the LMWH cohort. Re-thrombosis occurred in one patient of the LMWH group and none in the DOAC cohort. Re-bleeding in the time course of 1 year after craniotomy did not occur in both groups. We did observe a tendency to a reduced mRS at 6 and 12 months postcraniotomy and PE in the LMWH cohort; however, this was statistically non-significant (Fig. 1). Furthermore, patients in the DOAC cohort had a significant increase in the median OS with 15 months compared to 9 months at in the LMWH group (Fig. 2).

**Table 1:** Demographics of patients with glioblastoma and pulmonary embolism organized according to anticoagulation regime. Abbreviations: *mRS*, modified Rankin Scale; *DOAC*, direct oral anticoagulants; *LMWH*, low-molecular-weight heparin; *PE*, pulmonary embolism; *GBM*, glioblastoma; *SD*, standard deviation; *MGMT*, O(6)-methylguanine-DNA methyltransferase; *IDH-1*, isocitrate dehydrogenase 1; *KPS*, Karnofsky Performance Status; *IQR*, interquartile range

Characteristics	DOAC *n* = 14	LMWH *n* = 32	*p*-value
Male, *n* (%)	6 (43)	12 (38)	n.s
Median time from craniotomy to discharge in days (IQR)	14.5 (7.5)	14 (4.75)	n.s
Median time from craniotomy to PE in days (IQR)	7.5 (6.75)	8 (5.5)	n.s
Mean age at time of PE (SD)	65 (8)	68 (13)	n.s
Admission status			
mRS 0–2 (%)	13 (93)	22 (67)	n.s
Median KPS (range)	90 (60–90)	90 (70–100)	n.s
Discharge status			
mRS 0–2 (%)	8 (57)	15 (47)	n.s
Median KPS (range)	80 (30–100)	80 (30–100)	n.s
Comorbidities			
Coronary heart disease	0 (0)	4 (13)	n.s
Tobacco abuse	0 (0)	6 (19)	n.s
Hypertension	5 (36)	12 (38)	n.s
Hyperchelesterinamia	1 (7)	3 (9)	n.s
Diabetes Mellitus	0 (0)	6 (19)	n.s
Medication at time admission			
Antiepileptics	7 (50)	15 (47)	n.s
Antidepressants	1 (7)	3 (9)	n.s
Platelet aggregation inhibitors	0 (0)	5 (16)	n.s
Vitamin K inhibitors	0 (0)	0 (0)	
DOACS	0 (0)	0 (0)	
Clinical course			
Gross total resection	9 (64)	13 (41)	n.s
Postoperative re-bleeding	1 (7)	2 (6)	n.s
Re-operation	1 (7)	2 (6)	n.s
Resucitaition	1 (7)	1 (3)	n.s
Sequela (%)			
Re-thrombosis	0 (0)	1 (3)	n.s
Major intracranial bleeding	0 (0)	0 (0)	
Re-embolism	0 (0)	0 (0)	
Outcome (%)			
Median KPS at 6 months (range)	90 (50–100)	90 (40–100)	n.s
Median KPS at 12 months (range)	80 (60–90)	70 (50–90)	n.s
mRS 0–2 at 6 months (%)	7 (64)	10 (48)	n.s
mRS 0–2 at 12 months (%)	6 (75)	9 (45)	n.s
Median overall survival (months)			
	15	9	0.014
Histopathology			
MGMT methylated	6 (43)	9 (28)	n.s
IDH-1 mutated	0 (0)	1 (3)	n.s

## Discussion

The treatment of VTE in neurooncological patients remains challenging. The risks of recurrent thrombosis and ICH in this fragile cohort are important as they contribute to patients’ morbidity and mortality, may interfere with radiotherapy, and increase the risk of hospitalization [18]. Recent clinical studies paved the way for DOACs in the treatment of cancer-associated VTE; however, DOAC treatment was associated with the increased risk of ICH in non-neurooncological patients [10, 17]. Despite the lack of prospective clinical trials, the international clinical practice guidelines for the treatment and prophylaxis of VTE in cancer patients recommended anticoagulation for confirmed VTE in patients with primary brain tumors with LMWH or DOACs [2].

To date, one retrospective comparative cohort study analyzed the radiographic images of 53 GBM patients (among others) for all intracranial hemorrhage (ICH) events. The cumulative incidence of ICH at 12 months following initiation of DOACs vs LMWH showed that DOACs were not associated with an increased incidence of ICH relative to LMWH in this cohort. However, only scarce clinical data is available and the impact on survival was not studied.

In our cohort, although statistical non-significant, patients with LMWH had a higher incidence of cardiovascular comorbidities. In the current literature, medical comorbidities are a well-established risk factor for PE [7, 15]. However, why GBM patients with comorbidities were allocated to the LMWH cohort remains cryptic. The answer, at least in part, could lie in the fact that the LMWH cohort showed reduced mRS at 6 and 12 months. The increased incidence of comorbidities and the reduced mRS indicate that these patients were severely more affected by the underlying disease resulting in the possibility of reduced compliance and the necessity of nursing care support. Once established, and dose adjusted, the s.c. injection of LMWH could have appeared as the more reasonable anticoagulant of choice. At the same time, since DOACs for PE in GBM remained off-label, the assurance of a high compliance was necessary for this anticoagulation approach. Data on DOACs for PE in GBM patients are scarce and the discussion of these findings remains theoretical.

We observed a reduced percentage of favorable outcomes in the LMWH cohort at 6 and 12 months, which showed no statistical significance. Surprisingly, the difference in OS was highly significant with 15 months in the DOAC cohort vs. 9 months in the LMWH. This finding is challenging. The occurrence of PE as a predictor for poor survival in GBM is well described and the higher incidence of comorbidities and reduced mRS in our LMWH cohort aggravate the clinical course at least in part. Furthermore, although statistically insignificant, patients that received DOACs in our cohort had a higher amount of MGMT methylated GBMs and better resection status. The small sample size of our cohort could therefore veil the statistical significance of this potential confounder. However, one cannot exclude the possibility of an impact of DOAC treatment on glioma biology and the tumor micro-environment. Micro-thrombotic processes that lead to limited blood supply, anoxia, and tumor-necrosis are important prognostic factors in GBM [9, 14]. The influence of anticoagulation on the tumor micro-environment is speculative and requires further prospective clinical investigation.

Our study has several strengths and weaknesses. As a strength, our investigation is the first study on the outcome of DOAC vs. LMWH in PE and GBM. As of retrospective character, all participants were added after GBM diagnosis and PE with similar initial therapy consisting of temozolomide-based combination of chemo-radiotherapy. The obvious limitation is that this investigation was a single-center study and of retrospective design. As this study is of observational character, confounding, selection bias, reverse causation, and uncontrolled statistical error risk cannot be excluded.

## Conclusion

Therapeutic anticoagulation remains challenging in patients with GBM and PE as prospective data is absent and recent neurosurgical intervention usually regarded as a contraindication. In our analysis, DOACs showed a satisfactory safety profile and appear therefore as a reasonable pharmakon for therapeutic anticoagulation in this vulnerable cohort.

## Data Availability

Anonymized data can be provided upon request.
